# Overwintering Strategy and Mechanisms of Cold Tolerance in the Codling Moth (*Cydia pomonella*)

**DOI:** 10.1371/journal.pone.0061745

**Published:** 2013-04-17

**Authors:** Jan Rozsypal, Vladimír Koštál, Helena Zahradníčková, Petr Šimek

**Affiliations:** 1 Institute of Entomology, Biology Centre of the Academy of Sciences of the Czech Republic, České Budějovice, Czech Republic; 2 Faculty of Science, University of South Bohemia, České Budějovice, Czech Republic; USDA-Agricultural Research Service, United States of America

## Abstract

**Background:**

The codling moth (*Cydia pomonella*) is a major insect pest of apples worldwide. Fully grown last instar larvae overwinter in diapause state. Their overwintering strategies and physiological principles of cold tolerance have been insufficiently studied. No elaborate analysis of overwintering physiology is available for European populations.

**Principal Findings:**

We observed that codling moth larvae of a Central European population prefer to overwinter in the microhabitat of litter layer near the base of trees. Reliance on extensive supercooling, or freeze-avoidance, appears as their major strategy for survival of the winter cold. The supercooling point decreases from approximately −15.3°C during summer to −26.3°C during winter. Seasonal extension of supercooling capacity is assisted by partial dehydration, increasing osmolality of body fluids, and the accumulation of a complex mixture of winter specific metabolites. Glycogen and glutamine reserves are depleted, while fructose, alanine and some other sugars, polyols and free amino acids are accumulated during winter. The concentrations of trehalose and proline remain high and relatively constant throughout the season, and may contribute to the stabilization of proteins and membranes at subzero temperatures. In addition to supercooling, overwintering larvae acquire considerable capacity to survive at subzero temperatures, down to −15°C, even in partially frozen state.

**Conclusion:**

Our detailed laboratory analysis of cold tolerance, and whole-winter survival assays in semi-natural conditions, suggest that the average winter cold does not represent a major threat for codling moth populations. More than 83% of larvae survived over winter in the field and pupated in spring irrespective of the overwintering microhabitat (cold-exposed tree trunk or temperature-buffered litter layer).

## Introduction

The codling moth (*Cydia pomonella*) is a major insect pest of apples and several other fruits such as pears, apricots, and walnuts [1]. This species probably originated in Eurasia, but later spread around the world following the development of apple cultivation, and now occurs in most apple producing areas in the temperate zone (in both southern and northern hemispheres). Additionally, it has also been reported in subtropical and tropical countries [1], [2]. The economic consequences of this pest, and the still unresolved difficulties in the implementation of routine large-scale programs for its management in apple orchards [3], are the two main compelling reasons for scientific interest in this species.

Knowledge of pest bionomy is a pre-requisite for the development of strategies for practical management of its populations. The life cycle of the codling moth is well described [4]. It overwinters as a diapausing fifth instar larva in a loose cocoon that is spun either under the bark of apple trees or in the litter near the base of trees [5]–[7]. In Central European populations (which this paper focuses on), the pupation of post-diapause caterpillars occurs inside their cocoons during the relatively prolonged period from late April until end of June, with a broad peak during May. Such a protracted period of pupation is not only influenced by differences in microclimatic conditions in larval overwintering sites, but probably also reflects genetically fixed individual variations. Some larvae allegedly persist a whole year and postpone pupation until the next spring [5]. Adults emerge from pupae in a similarly protracted manner. The first adults can be seen by the end of May, with peak emergence of the spring generation usually in June, and late individuals may come out in July (or even the next year). Eggs are laid on fruits, or on the leaves near the fruit, and the caterpillars develop inside the fruits. The codling moth has five larval instars regardless of temperature conditions with the optimal temperature for larval development between 28 and 32°C [8]. In Central European conditions, most caterpillars of the spring generation directly enter diapause and overwinter (monovoltine life cycle) [5]–[7]. Only a part of the population may reach the stage of a fully grown last instar by middle of July (depending on local weather and the particular year), and these caterpillars pupate and give rise to the summer generation (partially bivoltine life cycle).

Notes on winter survival/mortality of diapausing codling moth larvae are relatively rare in the literature. Several studies report very high mortality (often close to 100%) caused by bird predation on the larvae that overwinter under the bark of apple trees [9]–[14]. Given such high rates of predation, it is rather surprising that, according to some authors, the overwintering sites under bark are preferred over litter layer [15], [16]. Survival rates in the litter layer were not, to our knowledge, assessed in detail but two papers reported no or very little survival in soil [13], [14]. In addition to bird predation, winter cold may be an important mortality factor. The fluctuations of ambient temperature are typically much higher on exposed tree trunks than in the buffered litter layer. Intuitively, bird predation and winter cold might be two factors that drive larval preference for overwintering sites in the litter over the tree trunk. Although there are numerous brief notes on cold hardiness in older literature [17, and references therein], physiological mechanisms were studied in only two populations (Pacific Northwest in the USA, and Middle East represented by Iran). Neven [17] concluded that overwintering larvae of the Pacific Northwest population of the codling moth are freeze-intolerant, with LT_50_ close to the average whole body supercooling point (SCP), which ranges between −22°C and −24°C. Moharramipour 's group arrived to a very similar conclusion working with Middle East populations of the codling moth [18]–[20]. Both groups reported that trehalose is a prominent metabolite present in overwintering caterpillars and discussed its potential contribution to the extended supercooling capacity during winter. While a positive correlation between supercooling capacity and trehalose concentration was mentioned in [20], no relationship was observed in [17]. No elaborated study is available on cold tolerance in European populations of *C. pomonella*.

The main objective of this study was to assess cold tolerance and estimate the mortality caused by winter cold in the larvae of a Central European population of the codling moth on tree trunks and in litter layer. The caterpillars were regularly sampled in the field throughout the overwintering period, and their survival either in supercooled or partially frozen state was assayed. In order to elucidate physiological mechanisms of cold tolerance in this species, we measured supercooling capacity, osmolality of body fluids, thermal hysteresis between melting and freezing points, fresh and dry mass, hydration, levels of total lipids and glycogen, and we also performed detailed metabolomic analysis of organic acids, amino acids, sugars, polyols and free fatty acids. Some of our findings, such as high survival rates in litter layer, partial freeze tolerance, and winter accumulation of complex mixture of sugars, polyols and amino acids, were previously unknown for *Cydia pomonella* larvae and we discuss them not only from the perspective of overwintering in this insect pest but also in a broader frame of principles of insect cold tolerance.

## Materials and Methods

### Insects

Fully grown caterpillars of the last instar of the codling moth, *Cydia pomonella* (Walsingham, 1897) [(synonym: *Phalaena Tinea pomonella* (Linnaeus, 1758)] (Lepidoptera: Tortricidae) were collected from apple tree alleys in the vicinity of České Budějovice (48°59′NW, 14°29′EL) in South Bohemia, Czech Republic. Approximately 200 circular bands (width 30 cm) made of corrugated cardboard were mounted on the apple tree trunks at the height of approximately 1.5 m above ground during May 2010. The fully grown caterpillars spontaneously spun inside the bands during their wandering from the tree crown down to the soil. The caterpillars were collected on six sampling occasions during 2010/2011: 20 July 2010, 6 September 2010, 11 November 2010, 10 January 2011, 8 March 2011, and 11 April 2011 (see Fig. S1 for graphical scheme of sampling dates). Larvae were transported to České Budějovice, stored outdoors overnight and processed the next morning.

Caterpillars from laboratory culture were used for supplementary experiments (will be described later). The insect culture originated from field-overwintered caterpillars collected in South Bohemia during March 2010. The larvae were reared on artificial diet as described earlier [21], [22] in the Sanyo MIR 154 incubators (Sanyo Electric, Osaka, Japan). Long day photoperiod (18L ∶ 6D, 18 h light ∶ 6 h dark), constant temperature of 25±1°C, and relative air humidity ranging between 60–70% was used to promote direct development without diapause. In order to induce diapause in fully grown last instar, the eggs and all larval instars were reared under short day photoperiod (12L ∶ 12D), while the other conditions were equal to the long-day situation.

### Analysis of cold tolerance

We analyzed cold tolerance in field-collected and laboratory-reared insects using several different approaches. In the field-collected insects, supercooling point (SCP) was determined in eight individuals for each sampling date using programmable thermostat Ministat 240-cc (Huber, Offenburg, Germany) in combination with temperature data logger TC-08 (Pico Technology, St. Neots, United Kingdom) as described earlier [23]. Based on our preliminary experiments, the assays of cold tolerance were set as follows: (i) survival in supercooled state was tested at −5°C/14 d; −15°C/7 d (conducted for each sampling date); and −19°C/3 d (tested only in the caterpillars that were sampled in January 2011); (ii) survival in partially frozen state was tested at −5°C/1 h (conducted for each sampling date); and at −15°C/1 h; −20°C/1 h; −30°C/1 h (tested only in January 2011). The cold tolerance assays were performed as described earlier [23]. Briefly, the supercooled larvae (groups of 10–40 larvae for each exposure and sampling date) were exposed to the cold inside dry-cellulose-lined plastic tubes placed in freezers Calex 50 (Calex, Zlaté Moravce, Slovakia), while the frozen larvae were wrapped individually (*n* = 8 larvae for each sampling date) into moist cellulose to which a small ice crystal was added in order to stimulate freezing. For freezing assay, the Ministat 240-cc was programmed for: (i) slow cooling from 0°C to −5°C at a rate of 0.04°C/min (freeze exotherms of individual larvae were recorded as evidence of ice formation in their body fluids); (ii) maintaining −5°C for 1 h and, (iii) re-warming from −5°C to +5°C at a rate of 0.04°C/min. After this cold exposure, the larvae were kept at 25±1°C, r.h. 60–70% and long day photoperiod until they either died or pupated. Pupation was taken as a criterion of survival.

In order to assess winter survival in semi-natural conditions, we used laboratory-reared diapause caterpillars that were randomly divided into five groups (i–v) when they reached wandering stage of the last (5th) instar. The larvae were allowed to spin in the strips of corrugated cardboard (20 cm long, 5 cm wide, curled up into a tube). Then, all groups were gradually cold-acclimated at successively decreasing temperatures of 20°C, 15°C, 10°C, and 5°C (each temperature maintained for 3 days) in Sanyo incubators under short day conditions. After the cold-acclimation, all groups were moved outdoors on the 3rd of October 2010. Group (i) (*n* = 151 larvae) was placed in litter bellow the apple tree on the surface of the soil, covered with approximately 10 cm layer of leaf litter, and was left there until the next spring. Groups (ii–v) were placed on a tree trunk (height 1.5 m, facing North). The temperatures on the tree trunk (height 1.5 m, facing North) and in the litter layer (Fig. S1) were monitored in 2 h-intervals using data loggers 175-T1 (Testo, Lenzkirch, Germany). The data loggers were placed close to the larvae. On the 5th of January 2011, groups (ii–v) were manipulated as follows:

group (ii) (*n* = 159) was left on a tree trunk;
group (iii) (*n* = 50) was moved to the laboratory, constant 0°C/DD (darkness);
group (iv) (*n* = 97) was moved to the laboratory, daily temperature oscillation 10°C/0°C (12 h/12 h, DD);
group (v) (*n* = 97) was moved to the laboratory, daily temperature oscillation 0°C/−10°C (12 h/12 h, DD).

On the 10th of March 2011, all five groups were moved to the laboratory and exposed to 25±1°C, r.h. 60–70% and long days until they either died or pupated.

### Mass, hydration, osmolality and thermal hysteresis

Fresh mass (FM) was measured individually in ten larvae for each sampling date using Sartorius balance with sensitivity of 0.1 mg. Dry mass (DM) was measured after drying the specimens at 65°C for 3 days and hydration was calculated from gravimetric data and expressed as mg water/mg DM. Osmolality of hemolymph was measured individually in ten larvae for each sampling date using a vapor pressure osmometer Vapro 5520 (Wescor, Logan, UT, USA) as described earlier [23]. Presence/absence of thermal hysteresis factors in hemolymph was determined as a difference between equilibrium freezing and melting points using Clifton Nanoliter Osmometer (Clifton Technical Physics, Hartford, NY, USA) as described earlier [24], [25].

**Figure 1:**
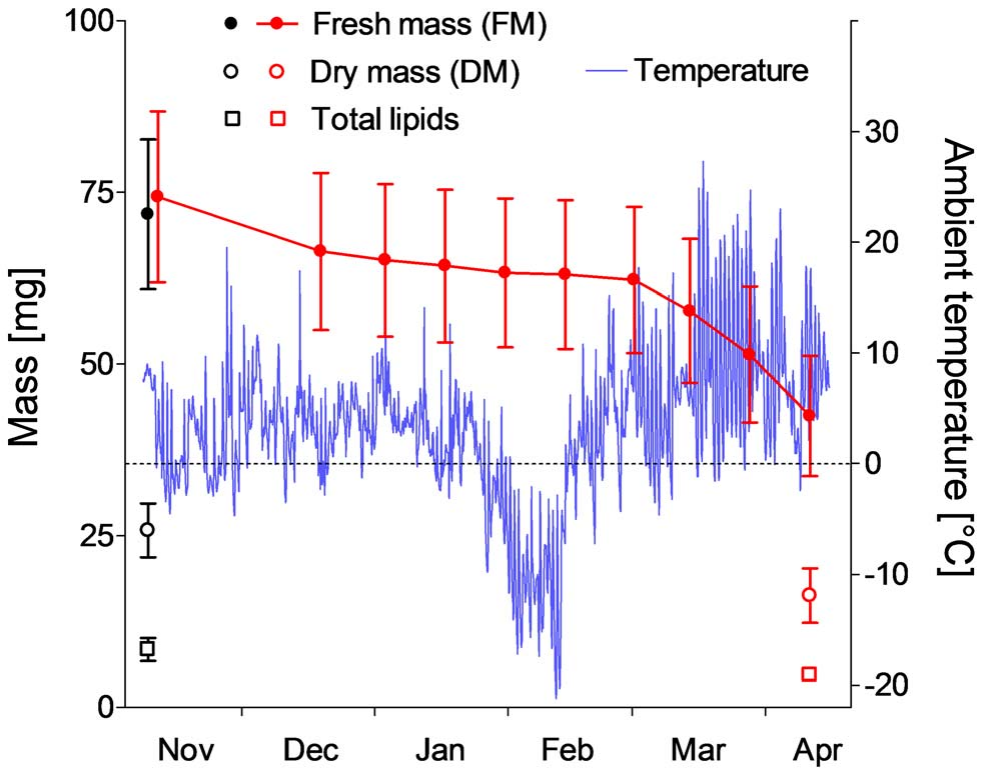
Fresh mass, dry mass, total lipid mass. Gradual losses of FM, DM and total lipid mass (all masses are in mg) in caterpillars of *Cydia pomonella* during their overwintering in the field in 2011/2012. Each point is the mean ± S.D. (*n* = 10 individuals). Black symbols are for larvae that were analyzed at the beginning of November, while red symbols are for larvae, in which gradual loss of FM was measured in approximately 14 d-intervals throughout the cold season and their DM and total lipids were analyzed in April (see text for details). The larvae were located on tree trunks (see text for details) and the course of ambient temperatures was recorded in 2 h-intervals.

In order to estimate loss of mass, water and lipid reserves in individual larvae overwintering in semi-natural conditions, we used laboratory-reared and cold-acclimated larvae (see above). In the first group of 10 larvae, FM, DM and total lipids were measured individually at the beginning of November 2011. In the second group of 10 larvae, only the FM was measured in November 2011 and the larvae were then moved outdoors in pre-weighed, punctured eppendorf tubes that were mounted on a tree trunk (see above). Gradual loss of FM was measured in approximately 14 d-intervals throughout the cold season 2011/2012. The course of temperature on the tree trunk (data logger Testo 175-T1) is presented in Fig. 1. The nine larvae that survived until spring were killed in the middle of April 2012 and their DM and total lipids were measured.

### Energy reserves and metabolomics

Total whole-body lipids were measured (five individuals for each sampling date) using spectrophotometric analysis with phosphoric acid-vanillin solution [26] after extraction of lipids by using chloroform∶methanol solution (2∶1, *v/v*) [27]. Whole-body glycogen content was measured in hemolymph (five individuals for each sampling date), and in two dissected tissues: body wall (epidermis with cuticle and muscle layer) and abdominal fat body. Tissues from three larvae were pooled together and three replications were prepared for each sampling date. Glycogen was extracted in hot alkali [28] and assayed using the colorimetric determination with phenol and concentrated sulfuric acid [29].

The metabolomic profiles were investigated in hemolymph, body walls and fat bodies pooled from three larvae (three replications for each sampling date) by a set of mass spectrometry-based methods as described earlier [23]. Briefly, the specimens were homogenized and extracted in 70% ethanol. Low molecular weight sugars and polyols were determined in ethanolic extracts after o-methyloxime trimethylsilyl derivatization and subsequent analysis by gas chromatography coupled to mass spectrometry (GC/MS). Additional profiles of free acidic metabolites (organic acids, amino acids, fatty acids) were obtained using a combination of GC/MS and LC/MS techniques in the same ethanolic extract after their treatment with ethyl chloroformate under pyridine catalysis and simultaneous extraction in chloroform. The concentrations of all metabolites were expressed in mmoles per 1 l of the respective water content in each tissue (*i.e.* mM).

### Statistics

One-way ANOVAs were used to analyze whether there is any influence of the sampling date on the measured physiological parameters. Bonferroni's post hoc tests were applied to find the differences among sampling dates. Unpaired two-tailed *t*-tests were used to assess the difference between the means of the two groups. Statistical calculations were performed using Prism v.4 (Graphpad Software, San Diego, USA).

The complex association of metabolomic changes as it related to the calendar season (sampling date) was determined by Principal Component Analysis (PCA) using Canoco v. 4.52 for Windows (Biometris-Plant Research International).

## Results

### Winter loss of mass, water and energy substrates

The non-diapause caterpillars of *C. pomonella* that were collected during July 2010 were relatively small (32.2 mg FM in average). The caterpillars that were collected during September 2010 (probably the next generation) were almost twice as large (62.1 mg) as the summer larvae and they entered into diapause. Table 1 summarizes changes of FM, DM and total lipids over the winter season 2010/2011. Although the field data clearly indicated that larvae lost FM during overwintering, the individual FM varied significantly probably influenced by non-random sampling bias (in addition to individual variation, females are larger than males). In order to obtain more precise data, we decided to repeat this measurement during the following season of 2011/2012, but tracking the gradual loss of FM in individual larvae (Fig. 1). At the beginning of November 2011, two groups of ten larvae showed practically equal mean FM (Student *t* = 0.4884, *P* = 0.6312), and the variances of the means were also statistically equal (*F* = 1.304, *P* = 0.3495). In November, the average FM was 74.4 mg; DM was 25.8 mg (38.9% FM) and the lipid content was 8.4 mg (11.7% FM). During the almost 6-month-long overwintering period until April 2012, the larvae displayed considerable losses of FM (average loss of 32.0 mg, *i.e.* 43.0% of initial FM), DM (10.45 mg, 39.1%), and total lipids (4.0 mg, 46.0%). Loss of water was calculated from gravimetric data (21.5 mg, 45.2%). Despite these considerable losses in absolute units, the relative contents of water and total lipids remained almost unchanged: water, 64.1% in Nov *vs.* 61.7% in Apr; total lipids, 11.7% in Nov *vs.* 11.1% in Apr.

**Table 1:** Seasonal changes in fresh mass, dry mass and total lipids in field-collected caterpillars of *Cydia pomonella*.

Sampling date	Fresh mass (FM)	Dry mass	Total lipids
	[mg]	[% FM]	[% FM]
July 2010	32.18±5.31 d	33.38±2.07 bc	13.33±1.71
September 2010	62.06±10.45 a	40.68±1.39 a	13.65±1.92
November 2010	48.97±7.49 bc	36.83±2.88 ab	11.97±0.93
January 2011	60.64±8.01 ab	38.48±4.23 ac	12.89±1.30
March 2011	40.77±8.43 cd	37.43±3.89 ab	12.17±3.19
April 2011	44.84±8.28 c	33.13±4.81 b	14.02±2.54
ANOVA *F*	16.22	5.883	0.7789
ANOVA *P*	<0.0001 (***)	0.0003 (***)	0.5746 (ns)

Data in columns (means ± S.D.) were analyzed using ANOVA (ns, no significant influence of sampling date on the parameter;

*** , highly significant influence). Means flanked with different letters are significantly different at *p* = 0.05 (Bonferroni's post-hoc multiple comparison test).

**Figure 2:**
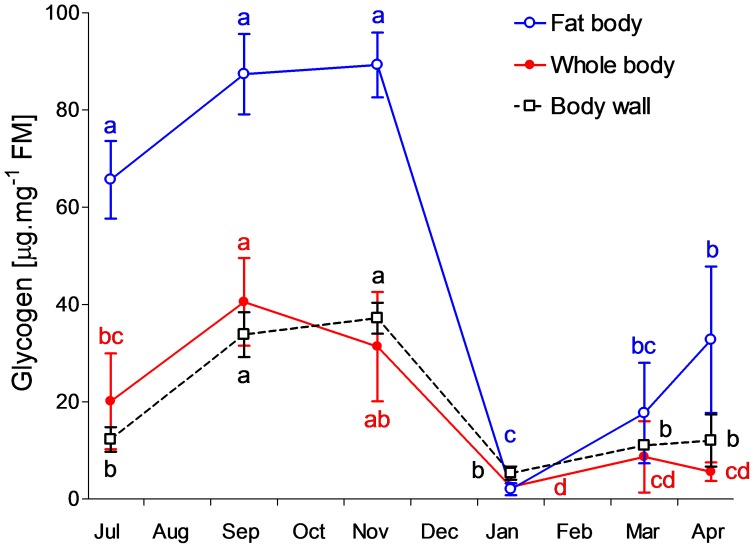
Glycogen. Seasonal whole-body and tissues changes of glycogen contents in field-sampled caterpillars of *Cydia pomonella* during 2010/2011. Each point is the mean ± S.D. (whole body, *n* = 5 individuals; tissues, *n* = 3 replicates, 3 individuals each). Influence of sampling date on glycogen content was tested by ANOVA followed by Bonferroni's post hoc test (means flanked with different letters are significantly different).

Whole-body glycogen content was approximately half in July-collected non-diapause caterpillars (20.1 µg mg^−1^ FM) when compared to September-collected caterpillars that were at the onset of their diapause (40.6 1 µg mg^−1^ FM). High levels of glycogen were maintained during the whole autumn. Massive depletion of practically all glycogen deposits was observed between November and January, both in the fat body and in the body wall, which is mainly composed of muscles. When the average FM of one caterpillar is considered as 50 mg, then approximately 1400 µg of glycogen reserves were depleted between November and January. Partial re-accumulation of glycogen was seen, at least in the fat body tissue, during the spring (Fig. 2).

### Winter accumulation of sugars, polyols and amino acids

**Figure 3:**
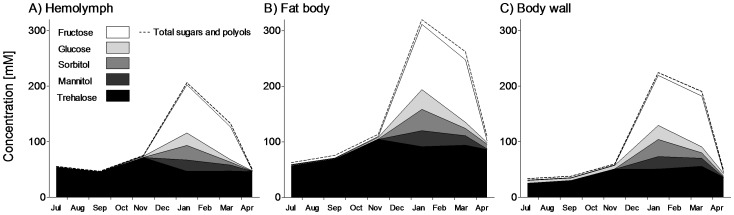
Sugars and polyols. Seasonal changes in concentrations of selected sugars and polyols in hemolymph (A), fat body (B), and body wall (C) of field-sampled caterpillars of *Cydia pomonella* during 2010/2011. The areas showing concentrations of individual compounds are stacked and the total concentration of all sugars and polyols is shown as a broken line. See Dataset S1 for details.

Fig. 3 depicts seasonal changes in concentrations of selected sugars and polyols. While the concentration of trehalose was relatively high and more or less stable, four specific compounds were accumulated during the cold season. These “winter sugars and polyols”, namely fructose, glucose, sorbitol and mannitol, appeared in high concentrations between November and January but were almost completely cleared between March and April. The seasonal patterns were similar for hemolymph (Fig. 3A) and tissues (Fig. 3B, C). The total mass of four winter sugars and polyols that were accumulated between November and January was calculated to be approximately 830 µg for an average individual (50 mg FM). This calculation indicates that depleted glycogen reserves (1400 µg) were converted mostly to four winter sugars and polyols.

**Figure 4:**
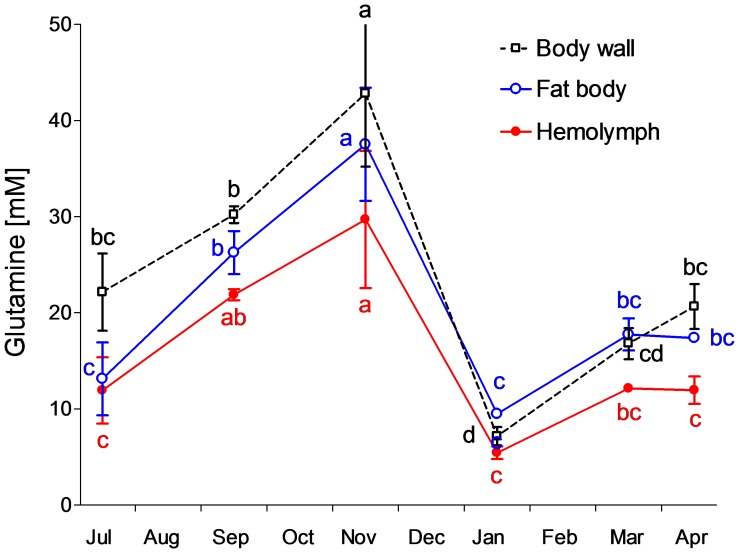
Glutamine. Seasonal whole-body and tissues changes of glutamine concentrations in field-sampled caterpillars of *Cydia pomonella* during 2010/2011. Each point is the mean ± S.D. (*n* = 3 replicates, 3 individuals each). Influence of sampling date on glutamine concentration was tested by ANOVA followed by Bonferroni's post hoc test (means flanked with different letters are significantly different).

**Figure 5:**
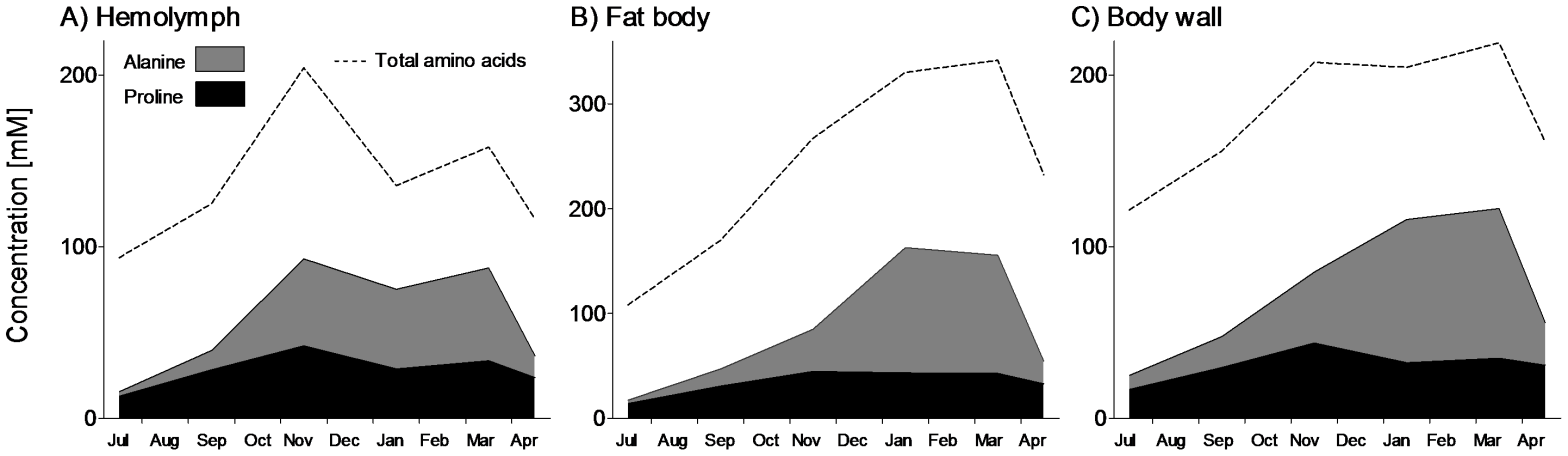
Free amino acids. Seasonal changes in concentrations of selected amino acids in hemolymph (A), fat body (B), and body wall (C) of field-sampled caterpillars of *Cydia pomonella* during 2010/2011. The areas showing concentrations of individual compounds are stacked and the total concentration of free amino acids is shown as a broken line. See Dataset S1 for details.

**Figure 6:**
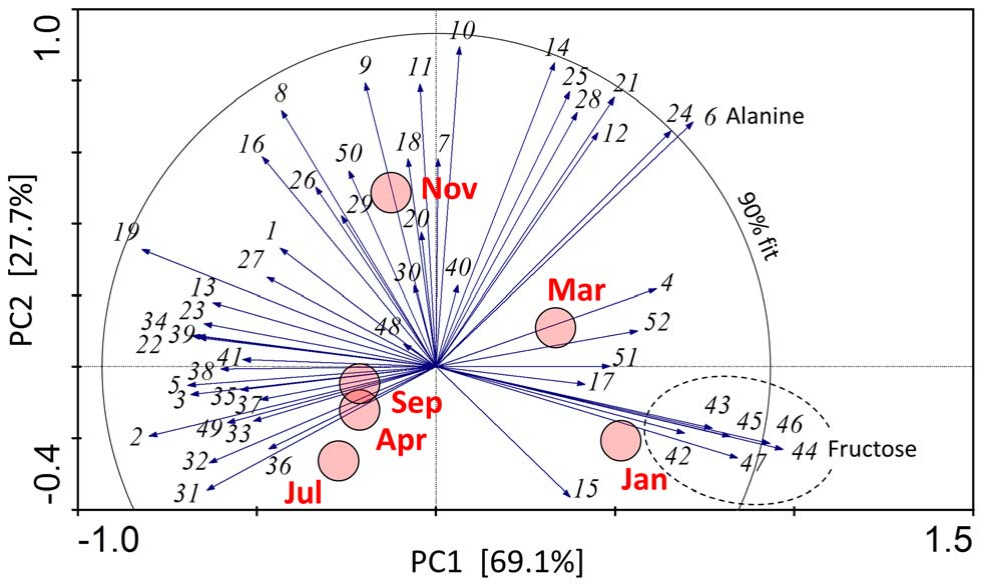
Hemolymph metabolom. Principal component analysis showing the association between sampling date (red circles) and the concentration of 52 different metabolites (eigenvectors) in the hemolymph of field-sampled caterpillars of *Cydia pomonella* during 2010/2011. The numbers coding for metabolites are decoded in Dataset S1. The eigenvectors of alanine (6), fructose (44), and mannitol (46) extend beyond the circle delimiting 90% fit of the model. The metabolites (42–47) most characteristic for winter (January) sample are enclosed by a dashed line ellipse.

Fig. 4 shows changes of glutamine levels in hemolymph and tissues. The seasonal pattern of glutamine concentration was similar to that of glycogen: the accumulation during autumn changed to massive depletion during the cold months and was followed by a partial re-accumulation during spring. Total pool of free amino acids increased during winter and alanine contributed most to the winter peak (Fig. 5). Alanine already significantly increased during autumn, between September and November (especially in hemolymph), reached a broad maximum during January–March, and was mostly cleared in April. Proline was the second most abundant amino acid and its seasonal pattern resembled that of trehalose - relative stability. In total, we followed changes in 52 different metabolites which are summarized in Dataset S1. Statistical analysis using PCA revealed that metabolomic compositions were similar in hemolymph samples taken in July (non-diapause), September (onset of diapause), and April (spring resumption of development) (Fig. 6). The sample taken in November (diapause maintenance/termination) was distinct from all other samples along the PC2 axis (explaining 27.7% of overall variance in metabolomic composition). This difference was driven by a group of compounds including valine (no. 9), leucine (10), isoleucine (11) and some others (Dataset S1). None of these metabolites, however, showed clear (statistically significant) seasonal change. Alanin (6) was also very high in hemolymph in November (Fig. 5A), but as this compound also remained high in January (end of diapause/beginning of quiescence) and in March (post-diapause quiescence), its eigenvector points to between Nov, Jan and Mar samples. The eigenvector of alanine is one of three eigenvectors, together with fructose (44) and mannitol (46), that extend beyond the circle delimiting 90% fit of the PCA model. Fructose and mannitol are two major compounds that drive the clear separation of January sample along the PC1 axis (explaining 69.1% of overall variance in metabolomic composition). Other characteristic metabolites contributing to January sample, namely glycerol (42); arabinitol (43); glucose (45), and sorbitol (47), are enclosed in an ellipse depicted by dashed lines (Fig. 6). The independent PCA analyses of the fat body and body wall metaboloms are presented in Figs. S2 and S3, respectively. Fructose and alanine were two compounds that systematically showed the most characteristic (winter-associated) and most significant changes in all three tissues (their eigenvectors always extended beyond 98% fit of the PCA model).

### Winter changes of supercooling capacity, osmolality and thermal hysteresis

**Figure 7:**
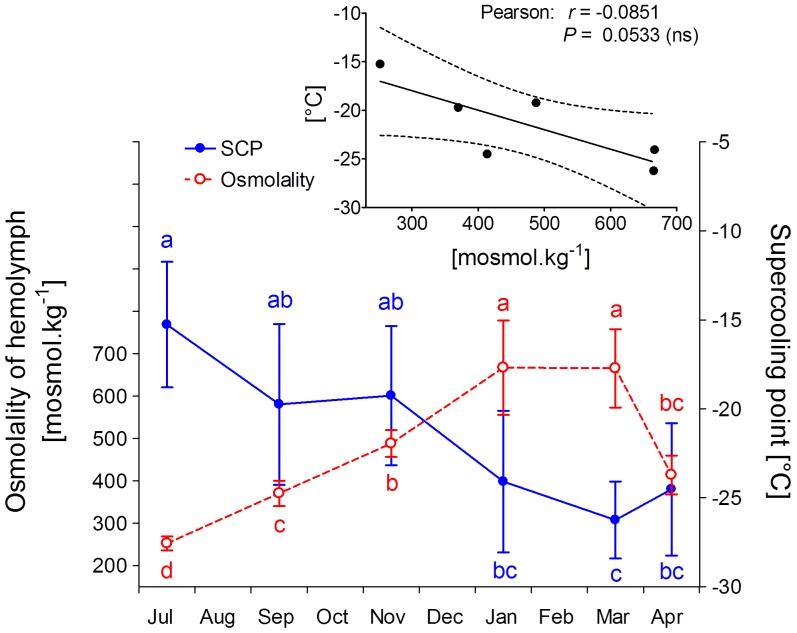
Osmolality and SCP. Seasonal changes of hemolymph osmolality and whole-body supercooling point (SCP) of field-sampled caterpillars of *Cydia pomonella* during 2010/2011. Each point is the mean ± S.D. (osmolality, *n* = 10 individuals; SCP, *n* = 8 individuals). Influence of sampling date on parameter was tested by ANOVA followed by Bonferroni's post hoc test (means flanked with different letters are significantly different). Inset shows that Pearson's correlation between osmolality and SCP was relatively tight (close to statistical significance).

The osmolality of hemolymph was relatively low (252 mosmol kg^−1^) in July-collected non-diapause larvae. In diapausing larvae, the osmolality gradually increased during autumn from 370 mosmol kg^−1^ in September to a broad maximum of 667–665 mosmol kg^−1^ in January–March, respectively. The April-collected larvae exhibited a slight decrease of osmolality to 414 mosmol kg^−1^ (Fig. 7). The correlation between hemolymph osmolality and whole body supercooling point (SCP) was close to statistical significance. Supercooling capacity was relatively low, which means that SCP was relatively high (−15.3°C), in the July-collected non-diapause larvae. The SCP gradually decreased with seasonal time, reached a minimum during March (−26.3°C), and also remained very low in the April-collected caterpillars (Fig. 7). The absolute minimum SCP (−28.4°C) was recorded in one caterpillar collected in March. None of the larvae in which SCP was measured survived freezing of body fluids.

No thermal hysteresis was detected in the non-diapause larvae. The least tiny ice crystal (approximate volume of 10 nL) began to grow immediately in the osmometer when the temperature was manually decreased by a single step of −0.0186°C (corresponding to a change of 10 mosmol kg^−1^). We detected extremely low hysteretic decreases of freezing point (ranging between 0.07°C to 0.11°C) in hemolymph samples of winter collected caterpillars (Table 2).

**Table 2:** Thermal hysteresis between the melting and freezing points in hemolymph samples taken from field-collected caterpillars of *Cydia pomonella*.

Sampling date	Thermal hysteresis^a^	
	[mosmol kg^−1^]	[°C]
July 2010	n.d.	0
September 2010	40	0.0744
November 2010	40	0.0744
January 2011	60	0.1116
March 2011	50	0.0930
April 2011	30	0.0558

a Difference between the equilibrium melting and freezing points was measured in a sample of hemolymph pooled from five individuals using Clifton Nanoliter Osmometer (n.d., no difference was detected).

### Winter increase of cold tolerance

**Figure 8:**
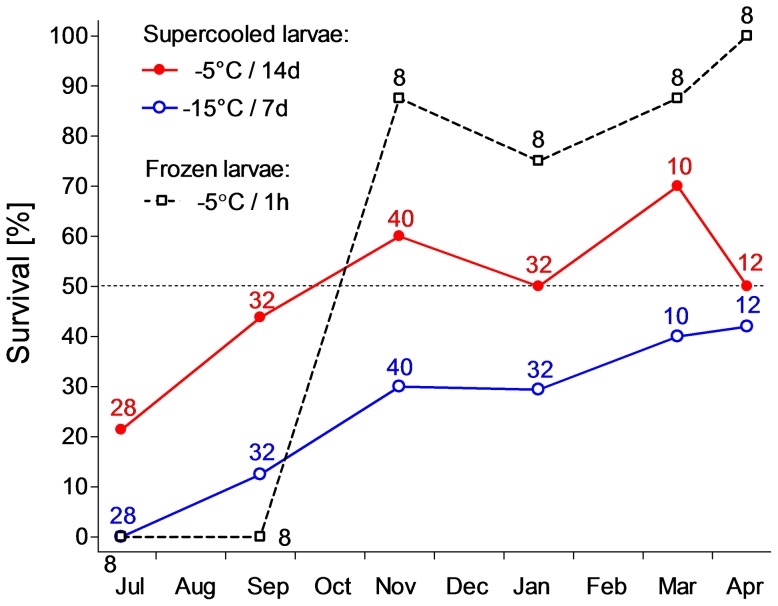
Cold tolerance. Survival at subzero temperatures in supercooled and partially frozen states in the field-sampled caterpillars of *Cydia pomonella* during 2010/2011. Each point is the percentage of survivors in a sample of *n* larvae (*n* = flanking number). Supercooled larvae were exposed either to −5°C for 14 d or to −15°C for 7 d. Partially frozen larvae were exposed to −5°C for 1 h.

The July-collected non-diapause larvae displayed relatively low capacity to tolerate subzero temperatures. None of them survived when exposed to −15°C for 7 d, and only 21.4% of them survived when exposed to −5°C for 14 d in supercooled state. No ability to tolerate freezing (−5°C for 1 h) was detected (Fig. 8). In diapausing larvae, the cold tolerance increased gradually with seasonal time and reached a broad plateau between November and April. Caterpillars collected between November and April mostly survived despite severe conditions of our survival assays. Thus, survival in supercooled state was approximately 50–70% for −5°C/14 d; 30–40% for −15°C/7 d (Fig. 8), and 36% in a single sample (January) exposed to −19°C/3 d (not included in Fig. 8).

The codling moth larvae were easily inoculated with external ice in our freeze-tolerance assays. The average onset of the larval body fluids' freeze exotherms occurred at −3.00°C±0.76°C (*n* = 48). The ability to survive after partial freezing of body fluids at −5°C was not observed in July- and September-collected larvae. Freeze-tolerance first occurred in November-collected larvae and, later, it stayed very high, ranging between 75–100%, until spring (Fig. 8). The ability to tolerate freezing to deep subzero temperatures (below −5°C) was assayed only in caterpillars that were collected in January 2011 (data not included in Fig. 8). We found that 25% larvae survived freezing down to −15°C/1 h, but no larva survived freezing to −20°C/1 h or to −30°C/1 h (*n* = 8 in each of the three treatments). All larvae that were used for our freezing assays were taken out of their cocoons prior to assay so that the external ice was in direct contact with larval integument during assay. In order to estimate the ability of a cocoon to prevent inoculation with external ice, we exposed 12 larvae (July-collected) to the same freezing assay but inside their cocoons. Only 4 of 12 cocooned larvae froze (and died), while 8 of 12 larvae supercooled (and 7 of those 8 survived until pupation).

Table 3 summarizes results of the whole-winter experiment of survival in various semi-natural conditions. Larvae mostly survived in all treatments. Fig. S1 displays the relevant records of microclimatic temperatures during winter season 2010/2011. The air temperatures fluctuated widely between +15°C and −15°C during the peak of winter (Dec, Jan), while the fluctuations were buffered to between +10°C and −5°C in the litter layer. Despite this difference, similar proportions of larvae survived on tree trunks (83.6%) and in the litter layer (86.1%).

**Table 3:** Winter survival in caterpillars of *Cydia pomonella* exposed to semi-natural conditions.

Group	(*n*)	Conditions^a^		Survival
		from 3 Oct 2010 to 5 Jan 2011	from 5 Jan 2011to 10 Mar 2011	[%]
(i)	151	outdoor, litter	outdoor, litter	86.1
(ii)	159	outdoor, tree	outdoor, tree	83.6
(iii)	50	outdoor, tree	lab, constant 0°C	72.0
(iv)	97	outdoor, tree	lab, 12 h 10°C/12 h 0°C	99.0
(v)	97	outdoor, tree	lab, 12 h 0°C/12 h −10°C	91.8

a Laboratory-reared caterpillars were used for this experiment. They were gradually cold-acclimated prior to transfer outdoors on 3 October 2010. Groups (i) and (ii) remained outdoors for the whole winter. Groups (iii–v) were moved back to the laboratory on 5 January 2011 and exposed to simulated winter conditions in incubators (under constant darkness). All groups were moved to constant 25°C on 10 March 2011 to check their survival (pupation). See text for more details.

## Discussion

### Potential impact of low temperatures on overwintering survival of codling moth population

In this paper, we extend considerably the knowledge of physiological principles of cold tolerance in overwintering larvae of *C. pomonella*, and bring new data to assess their winter survival. First of all, we would like to stress that both the earlier studies [17]–[20] and our own results suggest that low temperatures during the winter season do not represent a major threat for codling moth populations. This claim holds true, however, only when considering the conditions of an *average* winter. We observed high survival in our whole-winter 2010/2011 experiment conducted under various semi-natural conditions (Table 3). The larvae survived equally well in the litter layer (86.1% survival) and on tree trunks (83.6%). Survival on tree trunks was also high in 2012 (9 of 10 caterpillars survived) despite an exceptionally severe cold spell occurring in February 2012 (Fig. 1), during which the air temperatures remained below −10°C for almost 14 days and the night minima were close to or below −20°C.

The question remains open, however, what happens when an *extreme* winter comes. For instance, the historical minimum of air temperature in the Czech Republic was as low as −42.2°C. It was recorded close to České Budějovice (our study site) on 11 February, 1929 (data from the Czech Institute of Hydrometeorology). As we will discuss later, such temperatures would most probably kill 100% of codling moth population provided it overwinters in the exposed microhabitats of tree trunks. We believe, however, that larger parts of Central European populations prefer overwintering sites in the buffered microhabitat of the litter layer (see Fig. S1 for temperature differences between the two microhabitats). We mounted the cardboard bands on several hundred trees during this study. All trees (and many more in their vicinity) were carefully inspected for diapausing larvae prior to mounting the bands and during the winter samplings as well. Despite this effort, we found several specimens on old trees with deep scars in their bark or with loose bark scales. Our field observations contrast with data collected by Canadian authors [15], [16] suggesting that overwintering sites under bark are preferred over the litter layer and that survival in the litter layer is relatively low [13], [14]. Such discrepancy between our and Canadian data may be caused partly by differences in overwintering behavior between the distant populations of *C. pomonella*, and partly by differences in the “quality” of respective overwintering sites between the two countries. Surprisingly, the same Canadian authors reach a strong consensus that bird predation causes very high winter mortality (close to 100%) of codling moth larvae overwintering on trees [9]–[14]. Obviously, the question of preference for overwintering site requires additional field observations and tests. At least for South Bohemian populations of *C. pomonella*, the litter layer is preferred over tree trunks. We frequently observed that our cardboard bands were systematically destroyed by birds. Therefore, we assume that strong bird predation can be a major factor driving preference for overwintering in litter. As an additional benefit, the caterpillars that overwinter in the litter layer can safely overcome potential hazards of extremely cold winters.

### Strategy of freeze-tolerance in overwintering codling moth larvae

All earlier studies that were conducted in different parts of the world concluded that the overwintering larvae of *C. pomonella* rely on a strategy of extensive supercooling, which means avoiding the lethal freezing of their body fluids [17]–[20], [30]–[37]. Our study confirms supercooling as the main strategy of cold tolerance but also shows that the overwintering larvae of *C. pomonella* possess a good *physiological capacity* for freeze-tolerance. By observing the freeze exotherms in individual larvae, we have proven that the larvae were easily inoculated with external ice crystals at relatively high sub-zero temperatures (−3°C). They survived when partially frozen at temperatures down to −15°C (no survival was observed at temperatures of −20°C and −30°C). No survival, however, was observed in those larvae that froze spontaneously (*i.e.* without inoculation with external ice crystals) at relatively low sub-zero temperatures corresponding to their respective SCPs. It is well known, that most freeze-tolerant insects survive freezing only when the ice crystallization starts in the extracellular compartments at relatively high sub-zero temperatures and when the ice formation continues gradually, leaving necessary time for freeze dehydration of the cells and for the osmotic/ionic balancing across biological membranes [38]–[41]. Relatively high concentrations of trehalose (Fig. 3) and proline (Fig. 5) that were found in overwintering larvae might stabilize the structures of proteins and biological membranes during cellular freeze-dehydration [42]–[45]. We assume that the application of inappropriate assays for the evaluation of freeze-tolerance was the most probable cause why the capacity of freeze-tolerance was not described earlier in codling moth larvae. Thus, *C. pomonella* belongs to a growing list of insect species, in which careful analysis of cold tolerance revealed that they do not obey a strict dichotomy between the strategy of supercooling *vs.* freeze-tolerance but instead can employ both strategies [46]. However, we observed that the larval cocoons may represent relatively good, though not absolute, protection against external ice. In our assay, only 25% of cocooned larvae froze, while 100% of “naked” larvae froze. This observation raises the question of how much the strategy of freeze-tolerance is relevant under field conditions, where only cocooned larvae occur. Currently, we do not know. It is obvious, however, that both in the litter layer and on tree trunks the probability that an immobile cocooned larva comes in touch with external ice crystals (snow, hoarfrost, ice coatings on bark) is quite high. Thus, the capacity for freeze-tolerance can perhaps be considered in terms of ecological/evolutionary benefit (adaptation with positive effect on population survival), rather than in terms of a mere physiological capacity (with no real effect on population survival).

### Physiological principles of freeze-avoidance in codling moth larvae

In accordance with earlier studies [17]–[20], we conclude that freeze-avoidance (*sensu* [47], [48]), that is based on seasonal depression of SCP down to a minimum of −26.3°C, represents major physiological principle underlying high cold tolerance of overwintering codling moth larvae. No larva, either supercooled or frozen, was able to survive at temperatures below SCP, but on the other hand, many supercooled larvae survived long exposures to temperatures just above the SCP (either −15°C and −19°C in our laboratory assays or daily fluctuations between −10°C and −20°C in the semi-natural conditions during February 2012). The seasonal depression of SCP often comes in two steps in insects. The first step, which is concomitant with the entry into the diapause state, is linked to elimination/sequestering of the ice nucleators (mostly of unknown nature) from the gut and hemolymph [24], [49]–[51]. The second step is associated with cold acclimation and the accompanying buildup of high concentrations of solutes (cryoprotectants), which in turn affect colligative properties of body solutions including the water phase transition temperatures (equilibrium melting/freezing point) [52], [53].

We found that larvae of codling moth gradually lose water during overwintering (Fig. 1). This partial dehydration contributes to the increase of body fluids' osmolality that, in turn, correlates with the decrease of SCP (Fig. 7, inset). Accumulation of several metabolites, dominated by fructose (Fig. 3) and alanine (Fig. 5), represents an additional source of increasing osmolality/decreasing SCP in overwintering larvae. Fructose (and other sugars and polyols) most likely come from practically complete conversion of glycogen reserves, which is characteristically stimulated by low temperatures in the physiological context of limited need for energy turnover during deep diapause and low body temperature [53]. Alanine can originate partly from degradation of proteins (similarly to other amino acids found in winter larvae), but also partly from glycogen reserves. The end product of glycogenolysis, *i.e.* pyruvate, may be partially converted by alanine aminotransferase to alanine. The amino group for this reaction is provided by glutamate, which in turn, may be derived from decreasing reserves of glutamine (Fig. 4). Earlier studies [17]–[20] described the winter accumulation of trehalose in *C. pomonella*. Although the levels of trehalose were also high in our study, the seasonal changes were relatively small (Fig. 3).

Hemolymph osmolality increased from its minimum in July 2010 to its maximum in March 2011 by approximately 420 mosmol.kg^−1^. According to colligative law, this corresponds to a decrease of equilibrium melting point by −0.78°C, which may contribute to the depression of SCP by approximately −2.3°C to −4.7°C [24]. Storage proteins that typically accumulate in hemolymph of overwintering insects [54], including codling moth larvae [55], might further contribute to the decrease of SCP by non-covalently binding water in their hydration shells, thus diminishing the mobility of water molecules and their availability for formation of potential ice nuclei.

We detected some thermal hysteresis activity in hemolymph of winter-collected codling moth larvae. As this activity was extremely low in comparison to other insects (for review, see [56], [57]), we hesitate to speculate on its real biological relevance in particular case of codling moth. In other insects, active THFs counteract growth of ice nuclei by absorbing into the surfaces of small seed ice crystals [58] and, in this way they help to stabilize supercooled water and protect the insects from lethal freezing [59].

### On the role of cryoprotectants in the cold tolerance of codling moth larvae

Massive accumulations of fructose, alanine and some other metabolites were seen in codling moth larvae during the peak of the winter season (from November to March). High cold tolerance, however, outlasted these accumulations and persisted until late spring (April) when all of the typical cryoprotectants were cleared. In addition to rapid catabolism of these compounds, other signs of the spring resumption of development were observed in the April-sampled larvae: first, part of the population (approximately 10%) was already in pupal stage, and second, the larvae displayed relatively rapid loss of fresh mass, partial re-accumulation of glycogen and glutamine, and reversion of the trends in osmolality and SCP.

Such striking lack of positive correlation between the levels of “cryoprotectants” and the level of cold tolerance raises at least two important questions. First, what is the real relevance of the winter-accumulated compounds for cryoprotection? Second, what was the underlying mechanism of high cold tolerance in the April-collected larvae, if not high levels of cryoprotectants? Similar questions represent a recurring theme in the insect cold tolerance literature. Our current results, unfortunately, do now allow answering the questions with certainty. For instance, Denlinger [49] and Pullin [60] reviewed physiological relationships between diapause-related metabolic suppression, carbohydrate “cryoprotectant” biosynthesis and insect cold tolerance. They suggested that the diapause-related carbohydrate accumulation could be a primitive biochemical feature in ancestral (tropical) insect species, which evolved in linkage to their transition from active to dormant state (for review on cryoprotectant biochemistry, see [51]). In theory, such ancestral capacity might have later played an important source for natural selection during colonization of colder climates, where true cryoprotective roles of accumulated carbohydrates might be exploited (encaptation). In practice, tight statistical correlation between carbohydrate accumulation and high level of cold tolerance was observed in many overwintering insects [61]–[67], but not in all of them [68]. In the case of codling moth, high concentrations of fructose, alanine and other metabolites were not strictly needed to assure high survival, at least in our cold tolerance assays (note high survival in April when cryoprotectants were virtually absent). Despite this lack of correlation, we cannot simply conclude that the winter-accumulated compounds have no cryoprotective function under natural conditions. We would like to stress that the sum of accumulated cryoprotectants contributed significantly to the increase of osmolality and corresponding decrease of SCP. Since SCP is the absolute limit and a good indicator of cold tolerance in *C. pomonella*, the cryoprotectant-based depression of SCP by only a few degrees Celsius may be sufficient to protect a large part of overwintering population from lethal freezing. The SCP remained very low until April (−24.5±3.7°C). As our cold tolerance assays were conducted at −15°C, the April-sampled larvae were still safely above the temperatures that could stimulate stochastic occurrence of a lethal freezing event.

The high physiological capacity for freeze-tolerance in April-collected larvae is counterintuitive at the first sight. Nevertheless it was unequivocally confirmed in this study. Ongoing experiments in our laboratory aim to clarify the interconnected influences of seasonal water loss, changing cryoprotectant concentrations, and changing osmolality of body solutes on resulting amount of ice formed at a given temperature (*i.e.* −5°C). Our preliminary data suggest that significantly less amount of ice (in both, absolute and relative terms) is formed in the hemolymph of winter/spring-collected overwintering larvae than in the hemolymph of summer/autumn-collected larvae. We test the hypothesis that there is a critical amount of extracellular ice (or, vice-versa, a critical level of cellular freeze-dehydration), which, when exceeded, results in irreparable freeze-injury and larval mortality.

Our study confirms that seasonal acquisition of high cold tolerance is a highly complex phenotypic change, which involves numerous interplaying mechanisms. Further studies are needed to achieve higher level of knowledge, which could serve practical purposes such as the forecasting of codling moth populations' winter survival, timing of seasonal activity and outbreaks.

## Supporting Information

Dataset S1 **Concentrations of metabolites in hemolymph and tissues of field-sampled caterpillars of** ***Cydia pomonella*** **.**
(XLSX)

Figure S1 **Course of ambient temperatures in two overwintering microhabitats, tree trunk and litter layer, of the caterpillars of** ***Cydia pomonella*** **during 2010/2011.**
(DOCX)

Figure S2 **PCA analysis of metabolomic changes in the fat body of field-sampled caterpillars of** ***Cydia pomonella*** **.**
(DOCX)

Figure S3 **PCA analysis of metabolomic changes in the body wall of field-sampled caterpillars of** ***Cydia pomonella*** **.**
(DOCX)
