# Supplementary material for: Overwintering Strategy and Mechanisms of Cold Tolerance in the Codling Moth (Cydia pomonella)
Source: PLoS One. 2013 Apr 17;8(4):e61745. doi: 10.1371/journal.pone.0061745 (PMC3629207; doi:10.1371/journal.pone.0061745)
Supplement: Figure S1 — Course of ambient temperatures in two overwintering microhabitats, tree trunk and litter layer, of the caterpillars of Cydia pomonella during 2010/2011. (DOCX) [file pone.0061745.s002.docx]

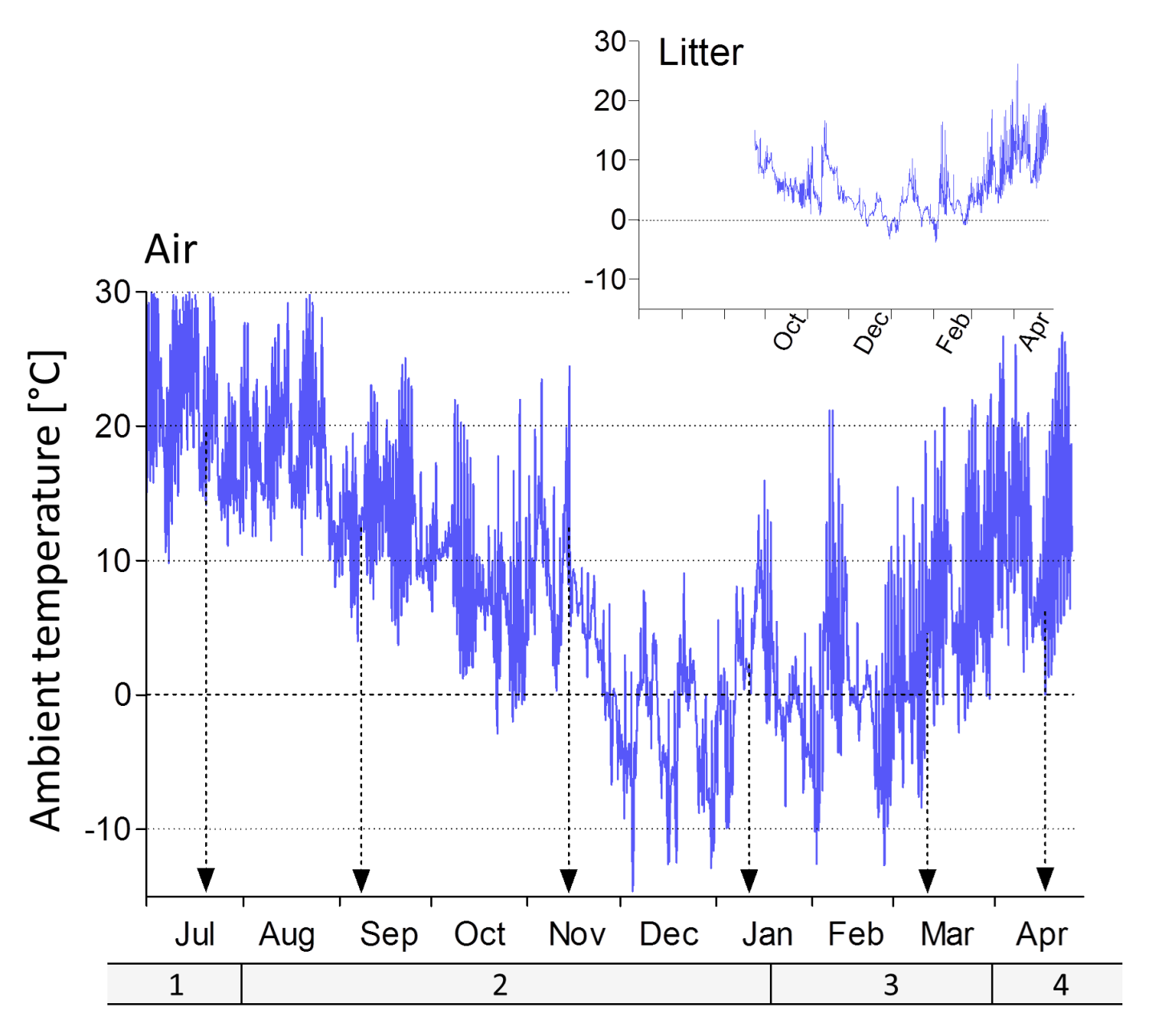


**Figure S1. Ambient temperatures.** Course of temperatures in two microhabitats (tree trunk and litter layer) where the caterpillars of *Cydia pomonella* overwintered during 2010/2011. Arrows show sampling dates. The bar below *x* axis schematically depicts duration of developmental phases: 1, non-diapause development; 2, diapause; 3, post-diapause quiescence; 4, spring resumption of development.
