# Supplementary material for: Overwintering Strategy and Mechanisms of Cold Tolerance in the Codling Moth (Cydia pomonella)
Source: PLoS One. 2013 Apr 17;8(4):e61745. doi: 10.1371/journal.pone.0061745 (PMC3629207; doi:10.1371/journal.pone.0061745)
Supplement: Figure S2 — PCA analysis of metabolomic changes in the fat body of field-sampled caterpillars of Cydia pomonella . (DOCX) [file pone.0061745.s003.docx]

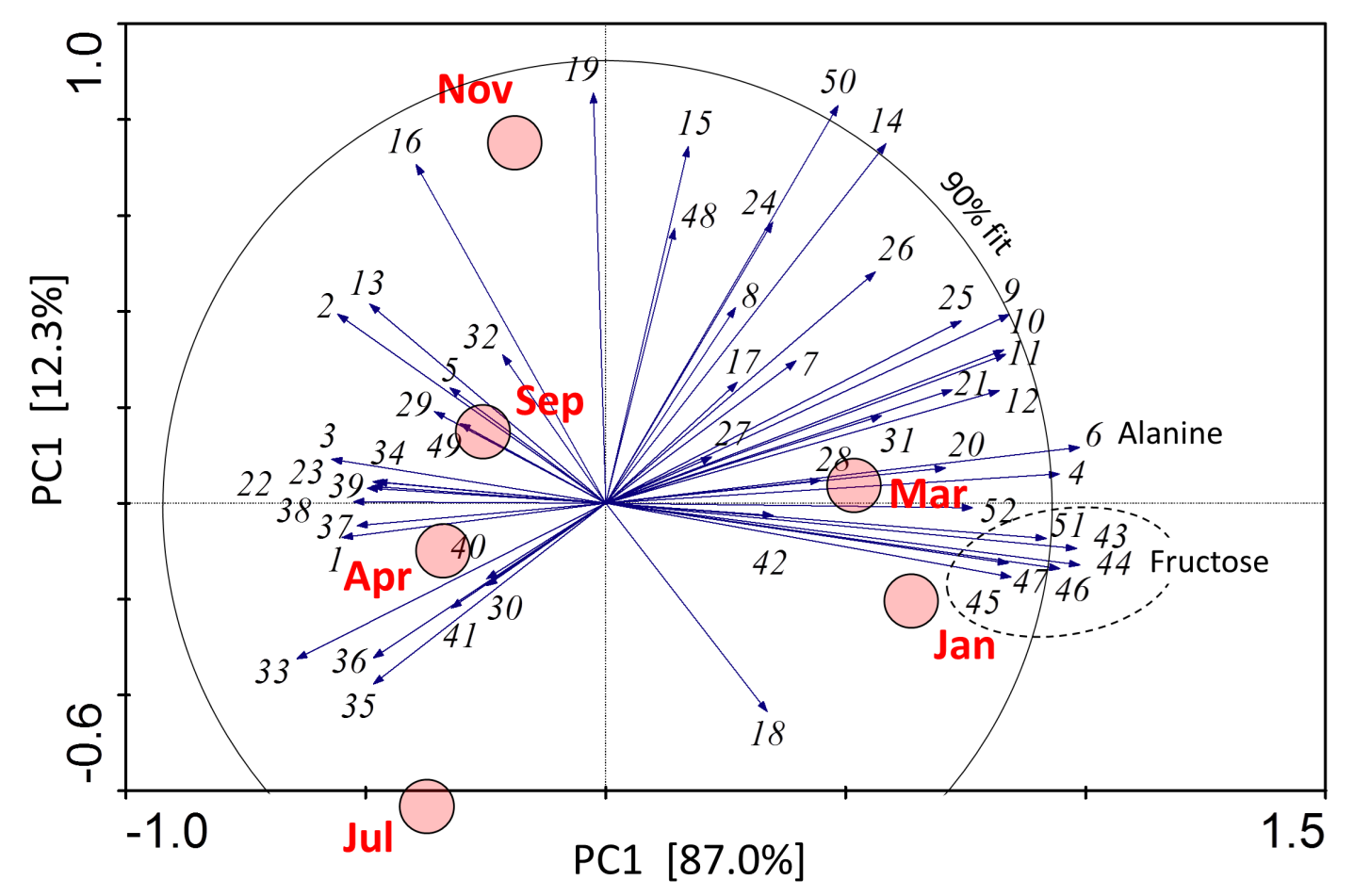


**Figure S2: Fat body metabolom.** Principal component analysis showing the association between sampling date (red circles) and concentrations of 52 different metabolites (eigenvectors) in the fat body of field-sampled caterpillars of *Cydia pomonella* during 2010/2011. The numbers of metabolites are decoded in Dataset S1. See Fig. 6 for more explanations.
